# Correlation between Different Patterns of Hypo-Osmotic Swelling and Sperm Functional Tests

**Published:** 2013-09-18

**Authors:** Farzaneh Bassiri, Marziyeh Tavalaee, Mohammad Hossein Nasr Esfahani

**Affiliations:** 1 Department of Reproductive Biotechnology at Reproductive Biomedicine Research Center, Royan Institute for Biotechnology, ACECR, Isfahan, Iran; 2 Isfahan Fertility and Infertility Center, Isfahan, Iran; 3 Department of Embryology at Reproductive Biomedicine Research Center, Royan Institute for Reproductive Biomedicine, ACECR, Tehran, Iran

**Keywords:** ICSI, HOST, DNA Fragmentation, Protamine

## Abstract

**Background:**

Sperm membrane integrity is not only important as a barrier between intra- and extra-cellular spaces, but also it can be considered as a sign of DNA integrity. Hypoosmotic swelling test reflects membrane integrity and has been used to evaluate sperm quality. Intracytoplasmic sperm injection (ICSI) in adjunct with hypo-osmotic swelling test (HOST) has been used for treatment of males with asthenozoospermia. Therefore, this study aims to evaluate correlation of different pattern of HOST with sperm parameters, protamine deficiency and apoptosis.

**Materials and Methods:**

In this case-control study, sixteen semen samples were randomly collected from infertile normozospermic men. Semen samples were divided into two portions as follows: one portion was assessed for sperm parameters according toWorld Health Organization (WHO)-2010, while the other portion, after applying HOST procedure, was used for assessment of sperm morphology, protamine deficiency and late or early apoptosis. Statistical analysis was carried out using the Statistical Package for the Social Studies (SPSS 11.5).

**Results:**

Our results showed that, the lowest odds ratio (OR) of abnormal sperm head morphology and abnormal acrosome was in d-sperm as compared to a-pattern or nonviable spermatozoa (p=0.00, p=0.01). In addition, a significant correlation was observed between d-sperm with sperm concentration and percentage of DNA damage (p=0.03 and p=0.04, respectively). A significant correlation was observed between percentage of sperm motility and DNA fragmentation (r=-0.56; p=0.01). Furthermore, significant correlations were observed between percentages of early apoptotic sperm with protamine deficiency and sperm concentration (p=0.009 and p=0.01, respectively).

**Conclusion:**

Significant correlations exist between d-pattern and sperm DNA integrity. Semen samples with low sperm concentration have low percentage of d-sperm which are mature and intact sperms.

## Introduction

The advent of Intracytoplasmic sperm injection (ICSI) was a fundamental and effective approach in domain of male infertility treatment (1). During ICSI, a viable sperm is selected based on its appearance and motility, and then, inseminated into a cytoplasm of mature oocyte. These two characteristics of sperm cannot solely ensure chromatin integrity of selected sperm (2, 3).

Considering the role of paternal DNA in development of sperm, selection of intact sperm for ICSI is of paramount importance (4-6).

An ideal sperm separation technique should present the following characteristic: i. remove abnormal and dead spermatozoa, leukocytes and bacteria, ii. abolish toxicants or bioactive factors [reactive oxygen species (ROS) and apoptosis], iii. be applicable to oligospermic samples, iv. avoid of provoking un-physiological ROS, v. separate live and motile sperm, vi. select functional sperm with intact DNA and membrane and vii. be a fast, simple and cost-effective procedure (7).

Recently, different novel sperm selection procedures have been proposed, for further details, see the reviews by Nasr-Esfahani et al. (8) and Said et al. (9), to achieve the above criteria based on special sperm characteristics or functional aspects. Due to failure of establishingthe efficiency of one technique over others, standard protocols and end points should be proposed to achieve a common consensus worldwide.

Hypo-osmotic swelling test (HOST),based on its subtypes, has been proposed as a simple, safe, and repeatable method in order to identify live and intact spermatozoa (10). Upon exposure of spermatozoa to hypo-osmotic condition, different tail patterns labeled from "a" to "g" according to World Health Organization (WHO) criteria are observed (11). This test is routinely used for diagnosis of male fertility potential, functional integrity of human sperm membrane (10).

Recently, Stanger et al. (12) have proposed this test for selection of intact spermatozoa and their findings have been further investigated by Bassiri et al. (12, 13). Therefore, Stanger et al. have proposed "d" or "e" patterns (12), while Bassiri et al. have suggested "d" or "c" patterns as the ideal sperms for insemination during ICSI procedure (13).

Previously, some studies reported a positive correlation between percentages of viable sperm, assessed by HOST,with sperm parameters, sperm zona-free hamster ovum penetration assay and in vitro fertilization (IVF) outcomes (14-16). However, in our recent study, we evaluated the percentages and frequencies of different sperm anomalies including: sperm abnormal morphology, DNA damage, apoptosis, and protamine deficiency in different HOST patterns (13). Therefore, this study is continuation of our previous study (13), and we aimed to evaluate the correlations between different HOST patterns with sperm parameters, protamine deficiency as sperm maturation marker, DNA fragmentation and early apoptosis in infertile normozospermic men.

## Materials and Methods

This case-control study received the approval of the Institutional Review Board of Isfahan Fertility and Infertility Center (IFIC) and Royan Institute. Sixteen semen samples of normozospermic men were collected from individuals attending the Andrology Unit of the Isfahan Fertility and Infertility Center, after signing a written informed consent document. 

### Sperm preparation and hypo-osmotic swelling test (HOST)

Sixteen semen samples were collected and each sample divided into two portions as follows: one portionwas washed with Ham’s buffer and used to assess sperm parameters according to World Health Organization (WHO)-2010, while the other portion was washed with Ham’s buffer. Then, 100 µl of the washed latter portion was diluted with 1 ml of hypo-osmotic swelling solution (Ham’s medium: sterile purified H2 O; v/v) at 37˚C for five minutes. Immediately, percentages of different patterns of HOST (a-g) were counted according to WHO criteria (11).

Accordingly, a-pattern was considered as nonviable sperm, while from b-pattern to g-patterns were considered as different degrees of alivesperm. Then, each HOS sample was put on a slide and evaluated for the following parameters: morphology, protamine deficiency, early and late apoptosis by Papanicoulau staining, chromomycin A3 (CMA3) staining, Annexin V staining and terminal deoxynucleotidyl transferase dUTP nick end labeling (TUNEL) assay. 

### Evaluation of abnormal morphology by Papani coulau staining

After HOST procedure, 20 µl of each sample was placed on a slide. Slides were manually stained by Papanicoulau staining according to WHO (11). 

Two hundred spermatozoa were counted for each sample and percentages of head and acrosome abnormalities were determined. Considering sperm tail curling during hypo osmotic swelling test and its effect on morphology of sperm tail and neck, we avoided reporting these abnormalities.

### Assessment of DNA fragmentation, protamine deficiency and evaluation of external phosphatidyl serine

After HOST procedure, 20 μl of each washed samples were fixed with paraformaldehyde and Carnoy’s solution for TUNEL assay and CMA3 staining, respectively, as described by Nasr-Esfahani et al. and Bassiri et al. For Annexin V staining, spermatozoa did not required to be fixed, so stainingwas carried out according to Bassiri et al. For each case, 200 spermatozoa were assessed randomly, and percentages of DNA fragmentation and CMA3-positive sperm were recorded (13, 17).

### Statistical analysis

Logistic regression analysis was used to obtain odds ratio (OR) for spermatozoa with abnormal head morphology and abnormal acrosome, then the obtained results were compared in each HOST subtypes with a-pattern sperm because this type of spermatozoa, as the worst type, was considered as reference group. Pearson correlation test was used to assess the correlations between parameters. Also, Statistical Package for the Social Studies (SPSS 11.5; Chicago, USA) software was carried out to analyze statistical data.

## Results

The mean sperm concentration was 69.46 ± 9.07×10^6^ spz/ml with a minimum and maximum of 7×10^6^ and 151.30×10^6^ spz/ml, mean sperm motility was 48.22 % ± 4.57 with a minimum and maximum of 40 to 70%, as well as mean sperm abnormal morphology was 85% ± 1.5 with a minimum and maximum 76 to 98%.

**Fig 1:**
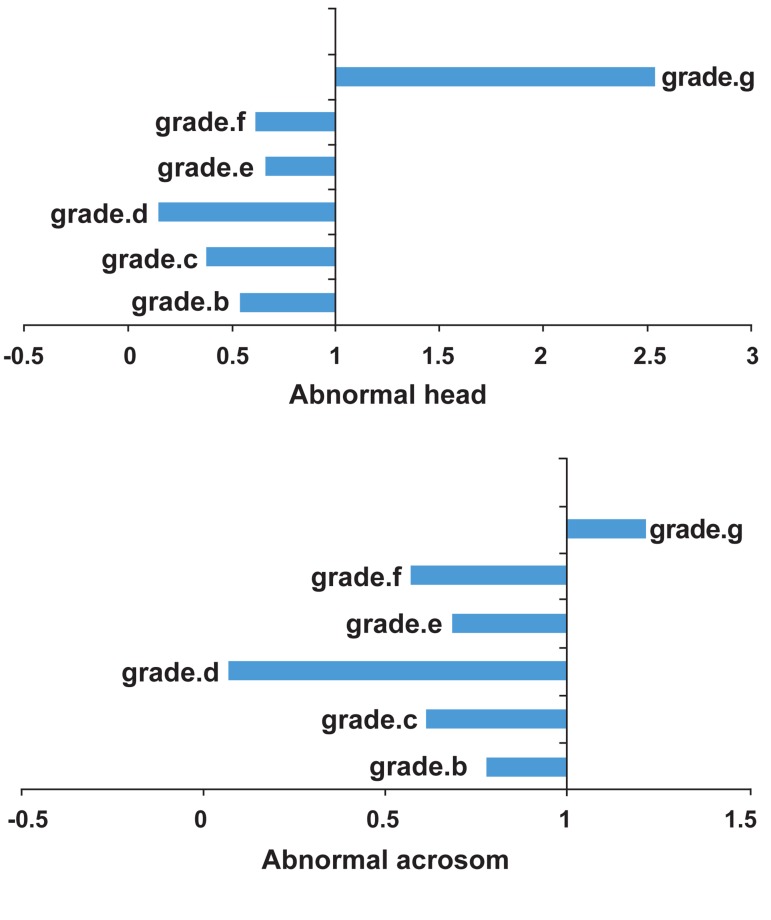
The ORs of abnormal sperm head morphology and abnormal acrosomefor HOST grade.

Hence, other patterns of HOS were compared with this reference group, and obtained percentage of abnormal sperm head morphology were presented as OR.The ORs for abnormal sperm head morphology of each HOST grades were as follows: 0.54, p=0.09 (b-sperm); 0.38, p=0.03 (csperm); 0.15, p=0.00 (d-sperm); 0.66, p=0.29 (e- sperm); 0.62, p=0.35 (f-sperm); and 2.54, p=0.05 (g-sperm) (Fig 1). The lowest OR of abnormal sperm head morphology belonged to d-sperm and c-sperm, respectively, which was significantly lower than a-sperm, while the highest OR belonged to g-sperm. Thus, the chance of confronting with an abnormal live g-sperm is 2.45 times higher than a necrotic or a-sperm.

The ORs for abnormal acrosome in spermatozoa of each HOST grade compared to a-sperm were as follows: 0.78, p=0.2 (b-sperm); 0.61, p=0.1 (csperm); 0.07, p=0.01 (d-sperm); 0.69, p=0.06 (esperm); 0.57, p=0.05 (f-sperm); and 1.21, p=0.18 (g-sperm) (Fig 1). The lowest OR for abnormal acrosome belonged to d-sperm which was significantly lower than a-sperm.The chance of abnormal acrosome in d-sperm was 12 times lower than in a-sperm, while the chance for g-sperm was higher than a-sperm, despite differences in the viability of sperm in these two groups.

 correlations between assessed parameters revealed a negative significant correlation between sperm concentration with protamine deficiency (r=-0.57; p=0.03) and percentages of apoptotic sperm (r=-0.664; p=0.01). In addition, a negative significant correlation was observed between percentage of sperm motility and DNA fragmentation (r=-0.56; p=0.01), while a positive significant correlation was observed between percentage of protamine deficiency and abnormal sperm morphology (r=0.59; p=0.02). Percentages of apoptotic sperm also showed a positive significant correlation with protamine deficiency (r=0.669; p=0.009) (Table 1). Assessment of correlation between sperm parameters, percentage of DNA fragmentation and protamine deficiency with different pattern of HOST (a-g) revealed a significant correlation between d-sperm with sperm concentration (r= 0.46; p= 0.03) and percentage of DNA fragmentation (r=- 0.46; p=0.04). In addition, a significant correlation was observed between b-sperm with percentage of sperm motility (r=0.5; p=0.02).

**Table 1:** Correlation between of conventional sperm parameters and percentage of DNA fragmentation, protamine deficiency and apoptotic sperm


Group	Sperm concentration (10^6^)	Sperm motility %	Abnormal morphology %	Apoptotic sperm %	DNA fragmentation %	Protamine deficiency %

**Sperm concentration (10^6^)**	1	0.410	-0.714**	-0.664**	-0.276	-0.574*
**Sperm motility % **	0.410	1	-0.329	0.048	-0.564**	-0.228
**Abnormal morphology % **	-0.714**	-0.329	1	0.496	0.182	0.598*
**Apoptotic sperm %**	-0.664**	0.048	0.486	1	-0.205	0.669**
**DNA fragmentation % **	-0.276	-0.564**	0.182	-0.205	1	-0.126
**Protamine deficiency % **	-0.574**	-0.228	0.598*	0.669**	-0.128	1


*; Show significantly different for p<0.05 , and **; for p<0.01.

**Table 2:** Correlation between of conventional sperm parameters, percentage of DNA fragmentation, protamine deficiency and apoptotic sperm with different patterns of HOST


Parameters	HOST a	HOST b	HOST c	HOST d	HOST e	HOST f	HOST g

**Sperm concentration (106)**	-0.343	0.358	0.103	0.466*	0.419	0.121	-0.049
**Sperm motility % **	-0.141	0.503*	0.433	0.116	0.152	0.089	-0.307
**Abnormal morphology center %**	0.350	-0.260	-0.057	-0.316	-0.268	0.015	-0.158
**Apoptotic sperm % **	0.328	-0.059	-0.018	-0.072	-0.234	-0.148	-0.417
**DNA fragmentation %**	0.192	-0.436	-0.361	-0.463*	-0.298	0.066	0.314
**Protamine deficiency % **	0.276	-0.325	-0.036	-0.237	-0.125	0.378	-0.383


*; Show significantly different for p<0.05 , and **; for p<0.01.

## Discussion

The structural and functional integrity of sperm plasma membrane is critical for the capability of spermatozoa for fertilization process. The most commonly tests for assessment of membrane integrity are eosin and HOST (10, 15). The eosin staining has provided information on sperm membrane structural integrity, while the While the HOS test has provided information on sperm membrane functional integrity. In other words, HOST assesses "ability of the sperm plasma membrane to transport water in hypo-osmotic condition, thus inducing tail swelling and plasma membrane stretching". Due to observation of seven forms of sperm tail, the classification are commonly referred to as "a" to "g" patterns (10, 18). Therefore, HOST has been considered as an easy, inexpensive, and reliable test for predicting male fertility potential and miscarriage rate in sub-fertile individuals (19, 20). In regard to this, since higher degree of swelling is easily observed in g-sperm, this pattern may represent the best sperm. This is in contrary to recent reports by Stanger et al. and Bassiri et al. evaluating sperm quality in different pattern of HOST (12, 13). These studies suggested that g-sperm presents higher degree of sperm abnormal morphology, DNA fragmentation, apoptosis and protamine deficiency. Our evaluation of sperm morphology revealed that the odd ratio for confronting an abnormal g-sperm is considerably higher than other pattern. In addition, based on our findings, OR for confronting an abnormal g-sperm is 2.5 times higher than the level of a-sperm or nonviable sperm, while OR for finding normal sperm is highest in d- and c-sperm. Similar results were observed when abnormal acrosome was assessed. Highest degree of normal acrosome was observed in d-sperm. Although we have no explanation for this observation, the below discussion may shed some light on this matter.

In this study, we did not observe a relation between sperm morphology with DNA fragmentation, despite a strong correlation between sperm morphology and protamine deficiency. In this regard, some authors suggested DNA fragmentation in spermatozoa with normal morphology is a proper selection for ICSI. Indeed, Avendan˜o et al. reported selecting a DNA fragmented sperm with normal morphology substantially increase in sub-fertile and infertile individuals (2). Taken together, these results suggest that the chance of selecting an apoptotic sperm and/or DNA fragmented sperm with normal morphology increases during ICSI in individuals with both low sperm concentration and motility. Therefore, other functional sperm characteristics for selection of sperm are required. According to our pervious study and findings of this study, the chances of confronting the best and worst spermatozoa are in d-sperm and g-sperm patterns, respectively (13). The correlations between different HOST patterns also reiterate this point. However, it is important to note that our obtained result should not be taken as a solid evidence for sperm integrity. 

The significant correlation between sperm concentration and DNA fragmentation with d-sperm suggest that percentage of d-sperm increases with sperm concentration, and the degree of sperm DNA fragmentation reduces in this type of semen samples. It is also interesting to note, the coefficient of correlations from (+0.5) decreases with increased tail swelling, and reaches a negative value (-0.3) in g-sperm. This suggests that there might be mechanism underlying the relation between motility and HOST patterns, which remains to be identified. One possible explanation for this observation might be functional distribution of Na^+/^K^+^ and Na^+/^H^+^ pumps in different patterns of spermatozoa. In reality, hypotonic resistance which is considered as a better term than HOST reflects the functions of these pumps in sperm plasma membrane (25).

It is likely that hyper distribution of these pumps might account for minimal swelling in b-sperm, while Na^+/^K^+^ ATPase dysfunction might account for g-sperm. ing-sperm. Therefore, optimal distribution of these pumps might be considered for best sperm, indicating that other functional properties in these spermatozoa are optimal, and this might be the reason for minimal sperm DNA fragmentation, apoptosis, proper histone/protamine exchange, etc. However, this hypothesis needs further evaluation.

## Conclusion

The results of this study suggest that there is a relation between sperm integrity and different HOST patterns. It is important to note, sperm membrane integrity can be relateddirectly to ROS production, but integrity of sperm membrane should not be considered as a direct indicator of DNA integrity, whereas DNA fragmentation can be observed even in sperm with normal morphology. The results of correlations in this study, specially the negative significant correlation between d-sperm and DNA fragmentation, verify the findings of the pervious study that d- and c-sperm should be selected for HOST-ICSI, while insemination of g-sperm should be avoided.
